# Histotripsy Outcomes for Primary and Metastatic Hepatic Disease

**DOI:** 10.3390/cancers18182931

**Published:** 2026-09-10

**Authors:** Elliott L. Fite, Nikhil Sekar, Mina S. Makary

**Affiliations:** Department of Radiology, The Ohio State University Medical Center, Columbus, OH 43210, USA; elliott.fite@osumc.edu (E.L.F.); nikhil.sekar@osumc.edu (N.S.)

**Keywords:** histotripsy, ablation, liver cancer, interventional oncology

## Abstract

Histotripsy is an emerging nonthermal, nonionizing, and noninvasive focused ultrasound therapy that uses acoustic cavitation to mechanically disrupt targeted tissue. In the liver, histotripsy may offer several advantages over conventional locoregional therapies, including treatment without percutaneous probes, treatment of select tumors near vascular or biliary structures, and potential proposed preclinical immunomodulatory effects. Early clinical studies, such as the THERESA and #HOPE4LIVER trials, have demonstrated high technical success rates and relatively low rates of major complications; however, expanding real-world evidence has identified clinically important complications including vascular injury, portal vein thrombosis, and acute kidney injury. Additional prospective studies are needed to determine long-term local tumor control, survival benefits, optimal patient selection, and the role of histotripsy in combination with systemic therapies and other locoregional treatments. This narrative review summarizes the possible mechanism of action, clinical outcomes, safety profile, patient selection considerations, and future directions of histotripsy for the treatment of primary and metastatic liver tumors.

## Simple Summary

Histotripsy is a novel, noninvasive treatment that uses focused ultrasound waves to destroy liver tumors without heat, radiation, or needles placed through the skin. Early studies have demonstrated that histotripsy can effectively target several types of liver tumors. Although initial studies reported relatively low rates of major complications, more recent clinical experience has identified important risks, including vascular injury, portal vein thrombosis, and acute kidney injury. While early results are promising, more research is needed to understand how well histotripsy controls tumors over time, which patients benefit most, and how it should be combined with other cancer treatments.

## 1. Introduction

Primary and metastatic liver tumors represent a major global oncologic burden and remain central to the practice of interventional oncology. In 2022, liver cancer accounted for 7.8% of global cancer deaths, making it one of the leading causes of cancer-related mortality worldwide [1]. Hepatocellular carcinoma (HCC) comprises the majority of primary liver cancers and typically arises in the setting of chronic liver disease, where treatment decisions are constrained not only by tumor burden but also by hepatic functional reserve [2,3,4]. Cholangiocarcinoma is the second most common primary hepatic malignancy, accounting for approximately 10–15% of primary liver cancers, and is frequently diagnosed at an advanced stage with limited curative options [5,6]. The liver is also the dominant site of metastasis for several common solid tumors, particularly colorectal cancer [3,7,8]. In these cases, hepatic disease burden often drives prognosis, eligibility for systemic therapy, and candidacy for potentially curative surgical or locoregional therapies [9,10,11].

Against this backdrop, the management of liver tumors has evolved into a multidisciplinary paradigm that integrates surgical resection, liver transplantation, systemic therapy, transarterial therapies, radiation-based approaches, and image-guided ablation [12]. For selected patients with early-stage HCC or limited metastatic disease, focal ablation can provide durable local control while preserving uninvolved liver parenchyma [13,14]. Radiofrequency ablation and microwave ablation are well-established modalities and are incorporated into contemporary treatment algorithms for patients who are not candidates for resection or transplantation [15,16,17]. Thermal ablation may be used with curative intent in selected patients with small HCC or CRLM. Randomized data from the SURF and COLLISION trials support ablation as an alternative to resection in appropriately selected patients [18,19]. However, conventional thermal ablation remains limited by several anatomic and biologic constraints [20]. Tumors adjacent to large vessels may be incompletely treated due to heat-sink effects, while lesions near bile ducts, bowel, gallbladder, diaphragm, or central vascular structures carry increased risk of collateral injury [14,21,22]. Radiofrequency ablation is particularly susceptible to perfusion-mediated heat sink effects near large vessels, whereas microwave ablation is generally less affected by convective cooling. Tumor size, visibility, location, and the ability to achieve adequate ablative margins further influence local tumor progression, particularly in metastatic lesions where wider margins may be necessary for durable control [22]. These limitations have created a need for nonthermal, liver-sparing technologies that can safely expand the population eligible for local tumor destruction.

Histotripsy is an emerging noninvasive, nonionizing, nonthermal focused ultrasound therapy that mechanically destroys tissue through controlled acoustic cavitation rather than heat, radiation, or percutaneous probe placement [23,24,25]. By generating high-amplitude ultrasound pulses at a defined focal zone, histotripsy produces cavitation bubble clouds that fractionate targeted tissue into acellular debris, creating sharply demarcated treatment zones while avoiding the temperature-dependent mechanisms that underlie thermal ablation [26]. This mechanism is clinically important because nonthermal mechanical disruption may reduce susceptibility to heat-sink effects and may permit treatment near vascular or biliary structures that would be high risk for radiofrequency or microwave ablation [24,27]. However, successful treatment remains dependent on adequate ultrasound visualization and a favorable acoustic window, and preservation of vascular and biliary structures should not be assumed. While collagen-rich structures appear relatively resistant to cavitation-mediated injury in preclinical and mechanistic studies, recent human studies have documented portal venous thrombosis, arterial injury, pseudoaneurysm formation, and clinically significant hemorrhage following hepatic histotripsy [28,29,30,31]. These findings emphasize that the nonthermal and noninvasive nature of histotripsy does not eliminate the potential for injury to structures within or adjacent to the treatment field and underscores the importance of careful patient selection, procedural planning, and post-treatment surveillance.

Histotripsy has recently emerged as a clinically available treatment option for liver tumors, representing the first noninvasive, nonthermal ablation platform approved for hepatic malignancies [32,33]. As the technology transitions from early clinical investigation into broader clinical practice, interest has grown regarding its potential role across a spectrum of primary and metastatic liver tumors [34,35]. Its major theoretical advantages, including noninvasive delivery, avoidance of thermal injury, potential treatment of lesions that are challenging for conventional thermal ablation, repeatability, and possible immunomodulatory effects, differentiate it from existing locoregional therapies and may expand treatment options for patients who are poor candidates for conventional ablation techniques [36].

Histotripsy may address the limitations of current liver-directed therapies by enabling nonthermal treatment without percutaneous applicator placement, particularly for selected tumors near vascular structures or where thermal ablation is limited. Despite this promise, the clinical role of histotripsy in liver oncology remains actively evolving [37]. Important questions remain regarding patient selection, disease-specific efficacy, optimal imaging response assessment, durability of local control, integration with systemic therapy, and comparative effectiveness relative to established locoregional treatments. Furthermore, the available literature encompasses a heterogeneous mix of prospective trials, retrospective studies, and early real-world experiences across diverse tumor types and clinical settings. This review synthesizes the current evidence for histotripsy in liver malignancies, with emphasis on disease-specific outcomes in HCC, cholangiocarcinoma, colorectal liver metastases, and other metastatic tumors, while highlighting the procedural, biologic, and oncologic questions that will define its future role in interventional oncology.

This manuscript was conducted as a narrative review. A literature search was performed using PubMed to identify studies evaluating histotripsy for primary and metastatic liver tumors. The final search was completed in June 2026 using combinations of the terms “histotripsy,” “liver,” “hepatocellular carcinoma,” “cholangiocarcinoma,” “colorectal liver metastases,” “liver metastases,” “focused ultrasound,” “ablation,” “clinical trial,” and “interventional oncology.” Preference was given to prospective clinical trials, multicenter studies, and contemporary clinical series. Relevant preclinical studies were included when necessary to describe the mechanism of action and emerging immunologic effects. Case reports, conference abstracts without peer-reviewed publication, non-English articles, and studies unrelated to hepatic applications were excluded. Additional references were identified through manual review of the bibliographies of included articles.

## 2. Mechanism and Procedural Principles of Histotripsy

**Figure 1:**
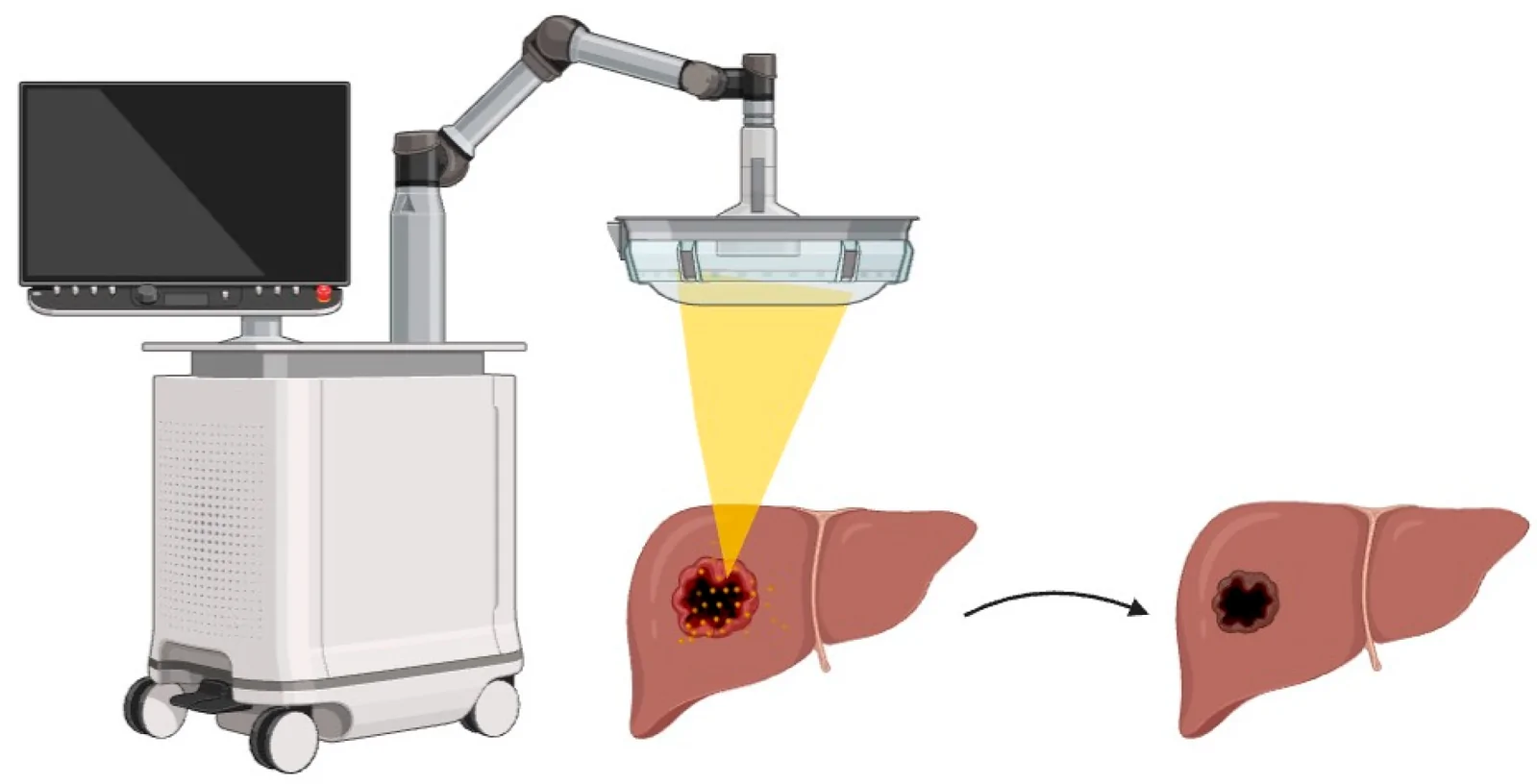
Schematic depiction of hepatic histotripsy. A robotic transducer delivers focused, high-amplitude ultrasound pulses to a targeted liver tumor, generating a cavitation cloud that mechanically disrupts tumor tissue without thermal injury. The treated lesion subsequently forms a well-demarcated treatment zone that can be assessed for residual tumor enhancement on post-treatment imaging.

Histotripsy is a noninvasive, nonionizing, nonthermal focused ultrasound ablation technique that destroys tissue through acoustic cavitation rather than heat (Figure 1) [38]. High-amplitude, short-duration ultrasound pulses generate a cavitation bubble cloud within the focal zone [39], and rapid bubble expansion and collapse mechanically fractionate tumor tissue into acellular debris while minimizing thermal injury (Figure 1) [40,41,42]. Because this effect is spatially confined to the acoustic focus, histotripsy can create sharply demarcated treatment zones under image guidance, while collagen-rich structures such as larger vessels and bile ducts demonstrate relative resistance to mechanical disruption in preclinical studies [23,24,38]. However, this differential tissue susceptibility is primarily supported by preclinical and mechanistic studies and should not be interpreted as evidence of universal vascular or biliary preservation in humans. More recent clinical experience has demonstrated portal venous thrombosis as well as arterial injury, pseudoaneurysm formation, and hemorrhage following hepatic histotripsy [30,31]. Accordingly, treatment near major vascular structures requires careful procedural planning and dedicated post-treatment imaging surveillance. Early liver studies, including THERESA and #HOPE4LIVER, have demonstrated the feasibility of this approach with high technical success and favorable early safety, although longer-term oncologic outcomes remain under investigation [28,29].

Histotripsy differs mechanistically from both high-intensity focused ultrasound (HIFU) and conventional thermal ablation [43]. HIFU is also extracorporeal and ultrasound-based, but its dominant mechanism is thermal coagulative necrosis generated by sustained tissue heating [44]. Similarly, radiofrequency ablation and microwave ablation destroy tumors through heat-based coagulation, while cryoablation relies on freeze-thaw injury; these modalities are therefore influenced by applicator position, heat transfer, tissue perfusion, and proximity to heat-sensitive structures [45,46]. In contrast, histotripsy is incisionless and nonthermal [47], making it less susceptible to heat-sink effects and potentially advantageous for tumors near major vessels or bile ducts, provided that the lesion is visible and an adequate acoustic window is available.

The biologic effects of histotripsy may also extend beyond focal tumor destruction. Preclinical studies have demonstrated local tumor suppression, improved survival, and systemic antitumor immune responses [26,48]. Mechanistically, mechanical tumor fractionation may release tumor-associated antigens, damage-associated molecular patterns, and inflammatory mediators in a manner that promotes immune activation while preserving antigenic structure more effectively than thermal coagulation [41,49,50]. In murine models, nonthermal histotripsy has been shown to stimulate tumor-specific immune responses and augment the effect of immune checkpoint inhibition [51]. These findings remain predominantly preclinical. Early studies have demonstrated changes in circulating cytokines and have described isolated imaging responses in untreated lesions; however, these observations are hypothesis-generating and do not establish a reproducible or clinically meaningful systemic antitumor effect. There is currently no prospective human evidence supporting selective treatment of lesions in patients with multifocal disease for the purpose of inducing immune-mediated control of untreated tumors. Therefore, intentional partial treatment or treatment of a subset of lesions for immune priming or an anticipated abscopal effect should be considered experimental and evaluated only within appropriately designed clinical trials.

## 3. Disease-Specific Clinical Outcomes in Histotripsy

Clinical outcomes after liver histotripsy remain preliminary, but available prospective trials, retrospective cohorts, and post-market registry studies demonstrate high technical success, relatively low rates of major complications in early studies, and emerging evidence of durable local tumor control (Table 1) [52]. Across studies, data are limited by heterogeneity in tumor histology, treatment intent, follow-up duration, imaging criteria, and whether lesions were treated completely or partially [34]. Nonetheless, disease-specific patterns are beginning to emerge for hepatocellular carcinoma, cholangiocarcinoma, colorectal liver metastases, and other metastatic liver tumors.

**Table 1:** Clinical evidence for liver histotripsy across prospective trials, retrospective cohorts, and real-world registry studies, highlighting study design, treated disease populations, efficacy outcomes, and safety profiles. Technical and oncologic outcomes are reported using the study-specific definitions, denominators, imaging modalities, and assessment time points provided by the original investigators. Successful targeting, technical success, technical efficacy, imaging evidence of complete treatment, LC, and LTP are distinct endpoints. ALT, alanine aminotransferase; AST, aspartate aminotransferase; CRLM, colorectal liver metastases; HCC, hepatocellular carcinoma; IDE, investigational device exemption; LOS, length of stay; LTP, local tumor progression; NED, no evidence of disease; OS, overall survival; PDAC, pancreatic ductal adenocarcinoma; POD, postoperative day.

Study	Design	Patient Population and Tumor Characteristics	Technical Outcomes	Clinical Outcomes	Safety Outcomes
THERESA [29]	Prospective, multicenter Trial	8 patients; 11 tumors HCC and metastatic liver tumors	Technical success in 11/11 tumors	LTP in 2/10 evaluable sites through 2 months	No device-related adverse events
#HOPE4LIVER [28]	Prospective, Multicenter Trial	44 patients; 49 tumors; HCC and metastatic liver tumors	Technical success in 42/44 (95%) tumors; 30-day imaging efficacy 38/46 (83%)	Long-term LC, LTP and OS not reported	Procedure-related adverse events in 7/44 patients (15.9%), including 3/44 (6.8%) major complications
#HOPE4LIVER 1-year update [53]	Prospective follow-up analysis	47 patients; 52 tumors; HCC and metastatic liver tumors	Technical outcomes not assessed	1-year LC 63.4% (primary review) and 90% (post hoc review); 1-year OS 73.3% for HCC and 48.6% for metastatic disease	6 serious device-related effects within 30 days; 1 nonserious device-related effect after 30 days
Chan et al. [54]	Prospective cohort study	30 patients; 60 tumors; 19 HCC and 11 metastatic liver tumors	36-h technical success 57/60 (95%); 1-month imaging efficacy 33/40 (82.5%)	Long-term LC, LTP, and OS not reported	Fever 13.3%, abdominal discomfort 10%; no grade III+ complications
Jimenez-Soto et al. [55]	Post-FDA retrospective cohort	27 patients; 42 tumors; mixed primary and metastatic liver tumors	Technical outcomes not assessed	No lesion growth within 3 months in 7/8 patients (87.5%); 3 downstaged to resection; 5 became transplant-eligible	Median LOS 19 h; minor complications in 41%; major events occurred in advanced/progressive disease
Mabud et al., liver metastases [56]	Retrospective cohort	26 patients; 56 metastatic liver tumors	Technical success in 54/56 (97%); no nodular enhancement in fully treated tumors at 1 month (*n* = 20/20) and 3 months (*n* = 5/5)	Long-term LC, LTP and OS not reported	Adverse events 15.3%; 1 grade 3 bacteremia; no 30-day deaths
Mabud et al., PDAC liver metastases [57]	Retrospective cohort	21 patients; 39 PDAC liver metastases	Technical success in 39/39 (100%) tumors	Long-term LC, LTP and OS not reported	2 minor adverse events; no major complications or deaths
Wehrle et al. International safety analysis [58]	Multicenter safety study	295 patients; 510 tumors, 18 centers. Safety-evaluable cohort: 230 patients, 9 centers	Technical efficacy not assessed	Long-term LC, LTP and OS not reported	Any complication 12/230 (5.2%); major complications 3/230 (1.3%)
Wehrle et al., first full year experience [59]	Prospective registry study	71 patients; 109 tumors; primary and metastatic liver tumors	No residual tumor on POD1 imaging in 60/82 (73%) complete-intent lesions	No residual tumor at 30 days (60/60) or 90 days (23/23); NED 70% after 1 treatment, 90% after retreatment	Complications 13%; 1 grade ≥ III event
Worlikar et al. [31]	Retrospective Safety Cohort	21 patients/21 tumors near or encasing major portal vein branches	Not assessed	Not assessed	Acute bland PVT 8/21 (38%); all resolved/improved with anticoagulation

Endpoint terminology varies across histotripsy studies and is reported according to the definitions and assessment time points used by the original investigators. Successful targeting refers to delivery of treatment to the intended lesion but does not necessarily indicate complete treatment coverage. Technical success refers to complete coverage of the planned target volume on immediate or early post-treatment imaging. Technical efficacy refers to the absence of study-defined imaging findings consistent with residual tumor at the first scheduled follow-up assessment. Local tumor progression indicates new or enlarging study-defined imaging findings consistent with tumor at the treated site after an initially successful treatment, whereas local control indicates freedom from local progression during follow-up.

### 3.1. Hepatocellular Carcinoma

For selected patients with early-stage HCC, resection or thermal ablation may be used with curative intent, while locoregional therapies may also support bridging, downstaging, or disease control. Histotripsy remains investigational, and its role relative to established curative therapies has not yet been defined. However, HCC has the strongest prospective clinical evidence for histotripsy, although long-term disease-specific outcomes remain limited. The first-in-human THERESA trial was a prospective, multicenter phase I feasibility study designed to evaluate technical success and safety in patients with primary and metastatic liver tumors [29]. This trial included only one patient with HCC, precluding meaningful disease-specific efficacy assessment. However, histotripsy successfully generated the intended treatment zone in all 11 tumors treated and was not associated with any device-related adverse events during the 2-month follow-up period [29]. Although the HCC-specific sample was small, the trial established feasibility of noninvasive mechanical ablation in a cohort that included both primary and secondary liver malignancies and provided early proof-of-concept data supporting further investigation of histotripsy as a treatment option for HCC.

The #HOPE4LIVER trial provided the pivotal prospective evidence supporting clinical adoption of histotripsy. This study consisted of parallel United States, European Union, and United Kingdom prospective, multicenter, single-arm trials evaluating histotripsy for primary and metastatic liver tumors [28]. Forty-four patients with 49 tumors were treated, including 18 patients with HCC and 26 with non-HCC liver metastases [28]. Technical success, defined as complete coverage of the targeted tumor volume by the planned histotripsy treatment zone on immediate post-procedural imaging, was achieved in 42 of 44 evaluable tumors (95%) [28]. Procedure-related major complications occurred in 3 of 44 patients (7%) within 30 days [28]. Although the primary report was not powered for HCC-specific efficacy, the inclusion of a substantial HCC subgroup established histotripsy as a technically feasible and safe liver-directed therapy in patients with primary liver cancer.

The 1-year #HOPE4LIVER update provides the strongest prospective evidence regarding the durability of histotripsy for HCC. In this follow-up analysis, 47 patients with 52 tumors were treated across 14 sites, including 19 patients with HCC [53]. Despite a population characterized by multifocal disease and advanced tumor burden, histotripsy demonstrated encouraging local tumor control. The overall 1-year local control rate was 63.4% by the primary core laboratory assessment and increased to 90% on post hoc review, suggesting that many lesions initially classified as treatment failures may have represented imaging interpretation challenges rather than true recurrence [53]. This discrepancy highlights the need for standardized imaging criteria when evaluating local control after histotripsy. Overall survival at 1 year was 73.3% among patients with HCC, supporting the potential clinical benefit of durable local disease control in this population [53]. These findings indicate that histotripsy can achieve sustained tumor control in selected HCC lesions and highlight the importance of accurate post-treatment imaging assessment when evaluating treatment efficacy.

Chan et al. further expanded prospective HCC-specific data in a single-center prospective cohort study of 30 patients and 60 tumors, including 19 patients with HCC and 11 patients with non-HCC liver tumors [54]. Eligibility required Child–Pugh A liver function and tumors less than 10 cm [54]. Technical success, defined as complete coverage of the planned treatment volume on post-procedural imaging, was assessed by contrast-enhanced MRI at 36 h, and treatment efficacy, defined by the investigators as complete radiographic response without imaging evidence of residual tumor within the treated lesion, was assessed at 1 month [54]. Among HCC tumors, technical success was 33/35 (94.3%), and 1-month efficacy was 27/33 (81.8%), similar to non-HCC tumors [54]. Procedure-related complications were mild, most commonly transient fever and abdominal discomfort, with no Clavien–Dindo grade III or higher complications [54]. This study also demonstrated a systemic cytokine response after histotripsy, including increased IL-6 and a four-cytokine panel that predicted complete response [54], suggesting a potential immunomodulatory effect that may be relevant to HCC treatment strategies combining locoregional therapy with immunotherapy.

Real-world HCC outcomes remain limited but generally support the findings of prospective studies. In the first full-year single-center prospective registry reported by Wehrle et al., 71 patients with 109 liver lesions underwent histotripsy, including seven patients with HCC [59]. Among lesions treated with complete ablation intent that demonstrated no imaging evidence of residual tumor on postoperative day 1, all maintained this imaging appearance at 30 days (60/60), and all lesions with available 90-day imaging remained without imaging evidence of residual tumor at that time point (23/23) [59]. Within the HCC subgroup, 6 of 8 lesions (75%) demonstrated no imaging evidence of residual tumor on postoperative day 1 [59]. These findings suggest that histotripsy can provide effective early local tumor control when complete treatment is achieved. However, the study also underscores the importance of treatment delivery and lesion accessibility, as only 60 of 82 lesions targeted for complete ablation demonstrated the study-defined imaging findings of complete treatment immediately after histotripsy [59].

Taken together, the available HCC data support histotripsy as a promising nonthermal locoregional therapy, particularly for small ultrasound-visible tumors and lesions located near major vascular or biliary structures where thermal approaches may be less desirable. Early prospective and real-world studies have demonstrated high technical success rates, favorable safety profiles, and encouraging short-term local control, suggesting that histotripsy may expand the therapeutic options available for patients who are poor candidates for thermal ablation or other liver-directed therapies. However, the current evidence base remains limited by relatively small patient numbers, short follow-up durations, heterogeneous treatment populations, and evolving post-treatment imaging assessment criteria. Additional studies are needed to better define long-term local control, recurrence patterns, survival outcomes, and optimal patient selection. Future research should also evaluate histotripsy in comparison with established therapies for HCC, explore its integration with systemic and immunotherapeutic approaches, and develop standardized imaging response criteria specific to mechanical ablation. As larger prospective studies and longer-term follow-up become available, the role of histotripsy within the multidisciplinary management of HCC will become more clearly defined.

### 3.2. Cholangiocarcinoma

Resection remains the principal curative treatment for localized cholangiocarcinoma. Ablative therapies are generally reserved for selected unresectable or recurrent disease, with the goals of local disease control or palliation, often alongside systemic therapy.

Clinical data for histotripsy in cholangiocarcinoma remain sparse and are largely limited to small clinical subgroups [60,61]. In THERESA, one patient with cholangiocarcinoma metastases underwent treatment of two tumors [29]. The study achieved acute technical success in all treated tumors and reported no device-related adverse events, but cholangiocarcinoma-specific local control and survival outcomes were not separately reported [29]. Therefore, THERESA supports technical feasibility rather than definitive disease-specific efficacy in cholangiocarcinoma.

Chan et al. included two patients with cholangiocarcinoma within a prospective cohort of primary and metastatic liver tumors. Although the study reported aggregate non-HCC outcomes rather than cholangiocarcinoma-specific endpoints, technical success and 1-month efficacy for the non-HCC group were 96.0% and 83.3%, respectively [54]. The study also included detailed examples of cavitation-zone involution, including an intrahepatic cholangiocarcinoma-HCC mixed tumor with marked reduction in treatment-zone volume at 1 month [54]. These findings suggest that histotripsy can be delivered to cholangiocarcinoma-containing lesions with early imaging response, but the number of patients is too small to define local control, durability, or survival.

Real-world post-market datasets provide additional evidence that cholangiocarcinoma lesions are being treated in practice. The international safety analysis by Wehrle et al. included 295 patients and 510 tumors treated across 18 centers after FDA clearance, with safety data available for 230 patients of the initial 295 cohort [58]. Of the 295 patients in the trial, 23 (8%) had primary cholangiocarcinoma [58]. The overall complication rate was 5.2%, with major complications in 1.3%, all attributed to disease progression in palliative patients rather than device-related injury [58]. However, efficacy was not assessed in this study, limiting its relevance to tumor control.

The first full-year single-center registry by Wehrle et al. included four patients with intrahepatic cholangiocarcinoma. In lesion-level analysis, 5 of 7 intrahepatic cholangiocarcinoma lesions (71.4%) demonstrated no imaging evidence of residual tumor on postoperative day 1 [59]. Because lesions demonstrating no imaging evidence of residual tumor on postoperative day 1 maintained this appearance at available 30- and 90-day follow-up, these early cholangiocarcinoma findings are encouraging [59]. However, the sample size is small, follow-up remains short, and outcomes are not yet comparable to established locoregional strategies. At present, histotripsy in cholangiocarcinoma should be framed as feasible and potentially useful in selected patients, particularly when tumors are adjacent to bile ducts or vessels where thermal injury is a concern, but disease-specific efficacy remains investigational.

### 3.3. Colorectal Liver Metastases

For limited CRLM, resection or complete thermal ablation may be performed with curative intent when all visible disease can be treated. In patients with unresectable or more extensive disease, local therapy is generally used with systemic therapy for disease control, conversion to resectability, or palliation.

Colorectal liver metastases (CRLM) represent the most extensively studied metastatic indication for liver histotripsy and comprise the largest metastatic subgroup across prospective and real-world series [62]. Current NCCN guidelines recommend multidisciplinary evaluation of patients with colorectal liver metastases to determine resectability and treatment intent. Complete surgical resection remains the preferred potentially curative approach when feasible, while ablation may be considered alone or with resection when all visible disease can be completely treated. More recent ESMO recommendations further reflect the evolving role of thermal ablation as a potentially curative local therapy in appropriately selected patients with limited CRLM [63]. Systemic therapy remains central for unresectable disease and may also be used perioperatively or to facilitate conversion to resectability [64].

Adequate ablation margins are critical for durable local control after thermal ablation of CRLM. Margins greater than 5 mm are generally recommended, while margins of at least 10 mm are associated with the lowest risk of local tumor progression [65]. More recent evidence from the randomized COVER-ALL trial and international multisociety Delphi consensus statements further supports quantitative, software-assisted assessment and standardized reporting of ablative margins to optimize local tumor control [66,67]. Importantly, the prospective OPTABLATE trial extended intraprocedural confirmation of treatment completeness beyond three-dimensional margin assessment alone. Treatment completeness was evaluated using three complementary endpoints: quantitative three-dimensional confirmation of an adequate minimal ablation margin; tissue sampling of the ablation zone and margin to demonstrate complete thermal artifact or absence of viable tumor using rapid histologic assessment, including mitochondrial viability staining; and real-time metabolic imaging with FDG PET/CT to confirm absence of residual FDG uptake within the region of the treated, previously hypermetabolic tumor [65,68]. Immediate additional ablation was performed when any of these assessments suggested incomplete treatment. This multimodal strategy emphasizes that confirmation of complete thermal ablation may require concordant geometric, tissue-based, and metabolic assessment rather than margin measurement alone [65].

Early CRLM outcomes with histotripsy are encouraging, but interpretation depends on whether complete treatment was achieved. In the first-in-human THERESA trial, 5 of 8 enrolled patients had CRLM, accounting for 7 of 11 treated tumors [29]. Although the study was designed primarily to assess feasibility and safety, all treated CRLM lesions were successfully targeted, and no device-related adverse events were reported [29]. During the 2-month follow-up period, local tumor progression was observed in 2 of 10 evaluable treatment sites; one was attributed to mistargeting related to poor ultrasound visualization, while the second occurred adjacent to untreated tumor, making true local treatment failure difficult to determine [29]. While durable CRLM-specific local control could not be established from this early feasibility study, the results demonstrated that histotripsy can safely achieve complete mechanical ablation of selected colorectal liver metastases and provided the foundation for subsequent studies evaluating longer-term oncologic outcomes in this population.

CRLM also represented a major component of the #HOPE4LIVER metastatic population. In the pivotal trial, 26 patients had non-HCC liver metastases, and the study achieved 95% technical success across primary and metastatic tumors, with 30-day technical efficacy of 83% among assessable lesions [28]. The 1-year update included 28 patients with metastatic disease, of whom 96.4% had multifocal hepatic tumors at the time of treatment [53]. While the study did not report CRLM-specific local control, 1-year overall survival for the metastatic cohort was 48.6%, and overall 1-year local control was 63.4% by primary assessment and 90% by post hoc review [53]. These data support local tumor control in a heavily pretreated metastatic population but should be interpreted cautiously because outcomes were pooled across metastatic histology.

Mabud et al. provide the most focused short-term imaging analysis of histotripsy for liver metastases. This single-center retrospective cohort included 26 treated patients and 56 metastatic liver tumors, with colorectal cancer as the most common primary tumor (32%) [56]. Overall technical success was 97% [56]. Fully treated tumors demonstrated favorable imaging characteristics, with all fully treated tumors with 1-month follow-up (20/20) and 3-month follow-up (5/5) showing hypoattenuation or hypointensity without nodular enhancement [56]. Partially treated tumors had more heterogeneous findings, often showing a treatment cavity adjacent to residual enhancing tumor [56]. Partially treated tumors demonstrated residual enhancing tumor adjacent to the treatment cavity. Incomplete treatment may result from inadequate visualization, targeting, acoustic access, or treatment coverage and should be considered technical failure rather than definitive ablation. [69,70]. In the same cohort, two patients demonstrated partial imaging responses in untreated tumors, including one patient with metastatic colorectal cancer [56]. Although these observations raise the possibility of systemic biologic effects, isolated responses in an uncontrolled cohort do not establish an abscopal treatment effect and should be considered hypothesis-generating. Intentional partial treatment for immune priming is not an established clinical strategy. There is currently no prospective human evidence demonstrating that incomplete treatment or selective treatment of lesions in multifocal disease results in clinically meaningful immune-mediated control of untreated tumors. Such an approach remains experimental and should be evaluated only within a clinical trial. Intentional partial treatment should also be distinguished from technically incomplete treatment, in which residual tumor is demonstrated on post-treatment imaging.

Jimenez-Soto et al. add clinically relevant data regarding CRLM in the post-FDA setting. This single-institution retrospective cohort included 27 patients and 42 tumors, with colorectal cancer representing the dominant pathology (17 patients, 63%) [55]. Histotripsy was used either for bridging/downstaging or palliation. Among the entire bridging/downstaging group, 7 of 8 evaluable patients (87.5%) had no lesion growth within 3 months; among palliative patients, disease control was lower at 33% [55]. Five patients with CRLM were treated with downstaging or bridging intent [55]. Histotripsy was used in some patients to limit liver progression during systemic therapy washout or before surgical management, and three patients with unresectable CRLM underwent transplant evaluation [55]. These findings position histotripsy as a potential adjunct in multimodal CRLM care, particularly when maintaining local control without interrupting systemic therapy is clinically valuable.

The largest real-world datasets also reinforce the relevance of CRLM. The international safety analysis from Wehrle et al. included 140 colorectal metastases and reported an overall complication rate of 5.2% among 230 evaluable patients, with major complications occurring in only 1.3% [58]. Most adverse events were Clavien–Dindo grade I or II, and no major complication was attributed to direct device-related injury despite treatment across 18 centers in routine clinical practice [58]. In the first full-year single-center registry, colorectal cancer was the most common diagnosis, accounting for 28 patients and 46 lesions [59]. On postoperative day 1, 26 of 46 CRLM lesions (56.5%) demonstrated no imaging evidence of residual tumor [59]. However, this also underscores the importance of targeting, because lesions with residual tumor identified on immediate post-treatment imaging represented incomplete treatment rather than late local failure.

Although the available CRLM experience is encouraging, the evidence base remains immature. Most reported patients have been treated within broader metastatic liver tumor cohorts, making it difficult to isolate disease-specific outcomes or determine how histotripsy performs relative to established approaches. Existing studies nevertheless suggest potential roles for histotripsy in bridging, downstaging, and local disease management when preservation of surrounding hepatic structures or minimization of treatment-related recovery is desirable. As clinical experience expands, dedicated CRLM investigations with longer follow-up, standardized response assessment, and comparative effectiveness analyses will be essential to clarify durability of tumor control, patterns of recurrence, and the most appropriate integration of histotripsy into contemporary multidisciplinary care pathways.

### 3.4. Other Metastatic Liver Tumors

Other metastatic liver tumors treated with histotripsy include pancreatic ductal adenocarcinoma (PDAC), breast cancer, neuroendocrine tumors [71], uveal melanoma, lung neuroendocrine tumors, sarcoma, and other less common histologies. Current evidence is most developed for PDAC liver metastases and mixed metastatic cohorts. Evidence for other liver metastases remains heterogeneous and is based primarily on small retrospective series.

Mabud et al. reported the first disease-specific cohort of histotripsy for PDAC liver metastases. This retrospective study included 21 patients with stage IV PDAC and 39 liver metastases treated at a single tertiary center [57]. Most patients (81%) were receiving concurrent systemic therapy, and many had extensive hepatic tumor burden, with 48% having more than 20 liver lesions [57]. Technical success was 100% across all treated lesions, and 81% of patients were discharged the same day [57]. Among 16 fully treated lesions, immediate ablation volumes were larger than baseline lesion volumes, and all fully treated lesions with 1-month follow-up demonstrated nonenhancing hypoattenuating or hypointense cavities [57]. At 3 months, 7 of 8 lesions had decreased in size without suspicious enhancement, and three fully treated lesions with at least 6 months of follow-up demonstrated 85–95% volume reduction [57]. Safety was favorable, with only two minor adverse events and no major complications or deaths. These data support the technical feasibility and short-term imaging response of selected PDAC liver metastases, although untreated lesions did not demonstrate objective off-target response, with 56% progressing and 44% remaining stable at 1 month.

Mixed metastatic liver tumor cohorts provide broader safety and imaging data across non-CRLM histologies. In Mabud et al.’s retrospective liver metastases cohort, the most common tumor types were colorectal (32%), breast (18%), biliary (14%), and neuroendocrine (11%) [56]. Fully treated metastases consistently demonstrated early nonenhancing treatment cavities without nodular enhancement at short-term follow-up, while partially treated lesions frequently showed residual adjacent enhancing tumor [56]. The study also reported favorable safety, with all patients discharged within 36 h, adverse events in 15.3%, one grade 3 bacteremia event in a patient with prior sphincter of Oddi violation, and no 30-day deaths [56]. This cohort demonstrated relatively low short-term morbidity in a mixed metastatic liver disease population, while emphasizing that treatment intent must be considered when interpreting efficacy.

The #HOPE4LIVER 1-year update included 28 patients with metastatic disease, including colorectal, breast, pancreatic, uveal melanoma, neuroendocrine, and other primaries [53]. Most patients had multifocal hepatic disease, and half had extrahepatic metastases [53]. While the results of the study suggest durable local treatment effect in a mixed metastatic population, they do not define histology-specific efficacy.

The post-market real-world studies expand this metastatic experience. In the international safety analysis from Wehrle et al., treated tumor types included colorectal metastases, neuroendocrine tumors, pancreatic tumors, breast metastases, cholangiocarcinoma, HCC, and other tumors. Safety data from 230 patients showed complications in 5.2%, with most being Clavien–Dindo grade I or II and no clear signal of device-related major toxicity [58]. In the first full-year single-center registry, metastatic tumors represented 81.6% of cases; neuroendocrine tumors accounted for 11 patients, breast cancer for 8, pancreatic cancer for 4, and other histologies for 9 [59]. On postoperative day 1, rates of no residual tumor identified on early post-treatment imaging were 66.7% for neuroendocrine tumors, 61.5% for breast cancer metastases, and 62.5% for pancreatic cancer lesions [59]. These rates reflect immediate imaging-based treatment effect rather than long-term local control, but the study found that lesions without imaging evidence of residual tumor on postoperative day 1 continued to so no evidence of residual tumor at subsequent 30- and 90-day imaging [59].

Across other metastatic tumors, histotripsy appears most compelling when used for ultrasound-visible lesions where nonthermal treatment, vascular and biliary preservation, minimal recovery time, and continuation of systemic therapy are clinically important. The available evidence supports feasibility, favorable safety, and encouraging short-term imaging response, but durable disease-specific efficacy remains incompletely defined. Future studies should stratify outcomes by histology, treatment intent, complete versus partial treatment, systemic therapy timing, and immunologic response to clarify which metastatic populations are most likely to benefit.

### 3.5. Conclusions for Disease-Specific Clinical Outcomes

Overall, current clinical evidence supports the technical feasibility of histotripsy across primary and metastatic liver tumors, with high early technical success and encouraging short-term imaging outcomes. The strongest prospective data are available in HCC, while studies in cholangiocarcinoma, colorectal liver metastases, pancreatic cancer metastases, and other metastatic tumors remain comparatively limited. Long-term disease-specific local control and survival outcomes remain incompletely characterized. Additionally, emerging post-market safety data demonstrate that clinically important vascular, thrombotic, renal, and infectious complications can occur and should be considered when interpreting the favorable safety findings of earlier clinical studies.

## 4. Safety Considerations and Patient Selection in Histotripsy

### 4.1. Safety Considerations in Histotripsy

Histotripsy avoids percutaneous applicator placement and thermal tissue injury, characteristics that contributed to an initially favorable safety profile in early clinical studies [28,29,72,73]. However, histotripsy is not risk-free. Reported and potential complications include bleeding, liver abscess formation, infection, transient post-procedural pain, nausea, fatigue, localized edema, and injury to adjacent structures if targeting is inaccurate [28,54,58,74]. In addition, transient post-procedural pain, nausea, fatigue, fever, infection, and injury to adjacent structures, expanding post-market experience has identified clinically important thrombotic, vascular, and renal complications [30,31,75,76].

Technical risks also include incomplete tumor ablation due to poor lesion visualization, respiratory motion, rib shadowing, excessive lesion depth, or obstruction of the acoustic pathway by bowel gas, lung, or bone [74]. In rare cases, patients may require additional procedures because of residual tumor or local recurrence identified on follow-up imaging [53,54]. Its safety depends on accurate ultrasound visualization, a safe acoustic window, avoidance of adjacent anatomic structures, and appropriate post-treatment imaging surveillance [28,74]. Absolute technical barriers include inability to visualize the lesion with ultrasound, absence of a safe acoustic window, and prohibitive interference from bowel gas, lung, or bone. Relative cautions include poor hepatic reserve, biliary colonization or active infection, coagulopathy, difficult tumor location, extensive disease burden, and uncertain clinical benefit. Vascular and biliary injury appear uncommon but remain possible, and careful patient selection and procedural planning are required.

Early clinical studies demonstrate a favorable short-term safety profile for histotripsy. In the THERESA trial, no device-related adverse events were reported during short-term follow-up, establishing the initial safety feasibility of hepatic histotripsy in humans [29]. The #HOPE4LIVER trial subsequently reported procedure-related major complications in 3 of 44 patients (7%), with no unexpected safety signals identified [28]. Longer-term follow-up from #HOPE4LIVER documented 6 serious adverse device-related effects within 30 days of treatment and only 1 nonserious adverse device-related effect beyond 30 days, suggesting that clinically significant device-related toxicity is uncommon and, when present, is most likely to occur during the early post-procedural period [53].

The largest multicenter post-market experience provides additional insight into patient candidacy and procedural risk in routine practice. In a safety-evaluable cohort of 230 patients, Wehrle et al. reported a low overall incidence of adverse events, with complications occurring in 12 patients (5.2%) and the majority consisting of low-severity events that required minimal intervention [58]. Higher-grade complications were infrequent and occurred predominantly among patients with extensive metastatic disease undergoing treatment for palliative purposes, suggesting that baseline disease burden and overall clinical status should be considered when evaluating risk and selecting appropriate candidates for histotripsy [58].

Metastatic liver tumor experience has demonstrated generally low rates of serious toxicity, although clinically significant complications can occur. Mabud et al. reported no 30-day mortality following treatment of liver metastases, with most patients recovering rapidly after the procedure [56]. The most notable adverse event was a case of grade 3 bacteremia that required extended hospitalization, emphasizing the potential for infectious complications despite the noninvasive nature of treatment [56]. This finding is particularly relevant in patients with prior biliary interventions, biliary-enteric reconstruction, recurrent cholangitis, immunosuppression, or other factors that may increase susceptibility to infection. Additional reported adverse effects were predominantly mild and self-limited.

Similarly, Chan et al. observed a low incidence of clinically significant toxicity, with no Clavien–Dindo grade III or higher complications reported during follow-up [54]. Reported treatment-related effects were generally minor and manageable, supporting the observation that histotripsy is associated with limited acute morbidity when performed in appropriately selected patients. Across published series, severe procedure-related complications have remained uncommon, and most adverse events have resolved with conservative management [54,58].

More recent clinical experience has identified acute bland portal vein thrombosis (PVT) as a clinically relevant complication when histotripsy is performed near major portal venous structures. Worlikar et al. evaluated 21 patients with tumors located within 10 mm of a major portal vein branch or with treatment zones encasing a portal vein branch and reported acute bland PVT in 8 of 21 patients (38%), all detected on immediate post-treatment imaging [31]. All thrombi resolved or improved with short-term anticoagulation without reported chronic hepatic imaging changes or clinical evidence of hepatic decompensation [31]. Sequential modification of the intraprocedural heparin protocol was associated with fewer and less severe thrombotic events, suggesting that anticoagulation may mitigate PVT risk [31]; however, the optimal prophylactic strategy has not yet been established or validated. These findings demonstrate that major portal venous branches remain susceptible to thrombosis during histotripsy and should be considered during treatment planning and post-procedural surveillance.

Clinically significant arterial injury has also been reported. Breuer et al. described vascular complications across four academic centers in which 4 of 139 patients (2.9%) developed serious vascular injuries following hepatic histotripsy [30]. All were identified on post-procedural contrast-enhanced CT, and three of the four patients required embolization for hemorrhage [30]. The reported complications included hemorrhagic and pseudoaneurysm-related vascular injuries and demonstrate that arterial wall injury can occur despite the nonthermal mechanism of histotripsy. These observations are particularly relevant when treating tumors abutting or incorporating hepatic arterial branches and argue against assuming universal vascular preservation. Post-treatment contrast-enhanced imaging should therefore be evaluated not only for treatment coverage but also for evidence of active bleeding, pseudoaneurysm formation, and other vascular injury. Similarly, Wong et al. reported two cases of arterial pseudoaneurysm following histotripsy for HCC, including one associated with a likely bilio-vascular fistula and another with portal vein thrombosis, further demonstrating that clinically significant vascular complications can occur following treatment [77].

Acute kidney injury has emerged as an additional potential systemic complication of hepatic histotripsy. Poe et al. reported a preliminary series of nine cases of acute kidney injury following liver tumor treatment [75]. The report raises concern that larger cumulative treatment volumes and multiple or overlapping treatment zones may contribute to renal risk. The underlying mechanism remains incompletely established and requires further investigation. These findings support assessment of baseline renal function, consideration of cumulative treatment volume, appropriate periprocedural hydration and medication management, and post-treatment renal function monitoring, particularly in patients undergoing larger-volume treatments or those with pre-existing renal risk factors.

Taken together, the evolving clinical literature demonstrates that the safety profile of hepatic histotripsy is more complex than suggested by the initial feasibility and pivotal trials. As emphasized by Sotirchos et al., the noninvasive nature of histotripsy should not be equated with absence of procedural risk [76]. Early prospective studies were relatively small and were not designed to characterize uncommon complications that may emerge with broader clinical use. Recent reports of portal venous thrombosis, arterial injury with hemorrhage or pseudoaneurysm formation, acute kidney injury, and infection indicate that risk may be influenced by treatment geometry, proximity to major vascular structures, cumulative treatment volume, baseline patient comorbidities, and other procedural factors [30,31,75]. Larger prospective registries with systematic adverse-event reporting are needed to establish the incidence and predictors of these complications and to define evidence-based strategies for prevention and surveillance.

### 4.2. Patient Selection in Histotripsy

Appropriate patient selection is essential because histotripsy remains an emerging liver-directed therapy, with strongest evidence in small, ultrasound-visible tumors and patients with preserved hepatic reserve [74,78]. In #HOPE4LIVER, eligible patients had primary or metastatic liver tumors, with up to three tumors of less than 3 cm selected for treatment [28].

Patient selection should occur through multidisciplinary review involving interventional radiology, hepatobiliary surgery, transplant surgery, hepatology, medical oncology, and diagnostic radiology [79,80]. Histotripsy may be considered for local tumor treatment, treatment of lesions poorly suited for thermal ablation, bridging or downstaging before transplant or resection, palliation, or adjunctive treatment in liver-dominant metastatic disease [81]. In each setting, the rationale for histotripsy should be compared with established options such as resection, transplantation, thermal ablation, SBRT, TACE, TARE, or systemic therapy.

The ideal candidate has preserved performance status, adequate hepatic reserve, limited liver-dominant disease, and a tumor that is clearly visible on ultrasound [82]. Because current clinical histotripsy is ultrasound-guided, inability to visualize the lesion is a major practical limitation [83]. Pre-treatment ultrasound should confirm lesion conspicuity, depth, respiratory motion, rib shadowing, intervening bowel gas, and feasibility of a safe subcostal or transcostal acoustic window [47,50,74]. Lesions in favorable anterior or inferior locations are generally more approachable, whereas superior dome lesions, deep posterior lesions, tumors obscured by ribs, and lesions near lung or bowel may be difficult or unsafe to target [74].

Hepatic reserve remains a key determinant of candidacy. Patients with Child–Pugh A liver function are best supported by current prospective data, while selected Child–Pugh B patients may be considered depending on tumor burden, treatment volume, transplant candidacy, and overall treatment intent [29,55,74]. Patients with Child–Pugh C disease, uncontrolled ascites, severe portal hypertension, active infection, or inability to tolerate anesthesia are generally poor candidates unless there is a compelling individualized palliative or bridging rationale.

Histotripsy may offer a nonthermal treatment option when conventional ablation is limited by tumor location [84]. Lesions adjacent to vessels, bile ducts, diaphragm, gallbladder, or other critical structures may benefit from a nonthermal approach [85]. However, recent reports of portal venous thrombosis, arterial injury, hemorrhage, and pseudoaneurysm formation demonstrate that proximity to vascular structures remains an important safety consideration [30,31,75,76]. Therefore, lesions adjacent to major portal or arterial branches should undergo careful individualized assessment rather than being considered intrinsically favorable for histotripsy solely because of its nonthermal mechanism. In metastatic disease, candidacy should also account for systemic disease burden, pace of progression, systemic therapy options, and whether local treatment of liver-dominant disease is expected to improve symptoms, preserve liver function, or contribute to broader disease control.

When selecting between histotripsy and established locoregional therapies, clinicians should consider not only tumor location but also lesion visibility, acoustic access, hepatic reserve, and treatment goals. Although histotripsy may offer advantages for tumors near vascular or biliary structures where thermal ablation may be challenging, it remains unsuitable for lesions that cannot be adequately visualized or accessed with ultrasound.

Relative contraindications include inability to visualize the tumor on ultrasound, lack of a safe acoustic window, intervening bowel or lung, extensive rib shadowing, excessive lesion depth, uncontrolled coagulopathy, active infection, high-risk biliary colonization, and unclear treatment intent [74]. Additional caution is warranted in patients with biliary stents, biliary-enteric anastomoses, recurrent cholangitis, immunosuppression, or tumors with extensive vascular or biliary involvement. Additional caution may also be warranted for tumors immediately adjacent to major portal or arterial branches and in patients with impaired baseline renal function or anticipated large treatment volumes. In summary, optimal candidates are patients with ultrasound-visible liver tumors, preserved hepatic and functional reserve, favorable acoustic access, and a clear oncologic rationale for choosing histotripsy over established liver-directed therapies.

## 5. Future Directions

Early clinical data support histotripsy as a feasible, noninvasive, nonthermal liver-directed therapy with high acute technical success and a favorable early safety profile; however, the field remains in an evidence-building phase, and recent post-market reports have identified important complications not fully characterized in the initial prospective trials. Current clinical evidence remains limited by small, predominantly noncomparative cohorts, short follow-up, variable imaging endpoints, and limited evaluation across broader patient populations. No prospective trials have directly compared short- or long-term outcomes of histotripsy with immunotherapy, TACE, or other established liver-directed therapies. Ongoing studies, including the BOOMBOX trial, are expected to provide broader real-world data on safety, workflow, patient selection, and treatment outcomes across multidisciplinary users.

Future studies must move beyond procedural feasibility and define the specific clinical scenarios in which histotripsy offers advantages over established locoregional therapies (LRTs), including tumors adjacent to vessels or bile ducts, patients with limited hepatic reserve, lesions poorly suited for percutaneous access, and patients undergoing bridging or downstaging to surgery or transplantation. The next phase of histotripsy research should therefore focus on real-world outcomes, combination strategies, biologic immune effects, technical optimization, and standardized imaging-response assessment.

### 5.1. BOOMBOX Trial

The BOOMBOX Master Study (ClinicalTrials.gov identifier: NCT06486454) represents an important step toward defining the real-world role of histotripsy in liver tumor management. Unlike the highly selected early feasibility and pivotal trials, BOOMBOX is designed as a prospective, observational, single-arm, nonrandomized master protocol evaluating the HistoSonics Edison System across multidisciplinary users and routine clinical practice [86]. BOOMBOX may provide broader real-world evidence regarding variation in procedural workflow, operator experience, patient selection, and outcomes across institutions that could not be evaluated in earlier controlled trials.

The study assesses histotripsy success with imaging within 36 h after treatment and follows patients according to standard clinical practice for up to 5 years [86]. This design is particularly valuable because it may capture broader variation in tumor biology, treatment intent, tumor location, acoustic window, body habitus, background liver disease, and procedural workflow than prior trials.

BOOMBOX may also help clarify several unanswered questions. First, it may identify patient- and lesion-level predictors of technical success, including tumor size, depth, proximity to ribs or lung, subcapsular location, and motion sensitivity. Second, it may help define whether early imaging findings after histotripsy reliably predict durable local tumor control, a key issue given evolving interpretation of post-histotripsy ablation zones and the discrepancy between primary and post hoc local control assessments in early follow-up studies. Third, the master-protocol structure may allow future sub-studies to evaluate disease-specific outcomes, including HCC, cholangiocarcinoma, colorectal liver metastases, pancreatic cancer liver metastases, and other secondary liver tumors. Finally, BOOMBOX may provide the practical data needed to determine how histotripsy should be integrated into multidisciplinary liver tumor boards, including whether it is best used as definitive local therapy, a complement to systemic therapy, a bridge to transplant, or a palliative cytoreductive tool.

### 5.2. Combination Therapies

#### 5.2.1. Combination Immunotherapy

A major future direction for histotripsy is its potential synergy with immunotherapy. Unlike thermal ablation, histotripsy mechanically disrupts tumor tissue, potentially preserving tumor antigens and promoting a more immunogenic response [49,51,87]. Preclinical studies have demonstrated immune activation, increased immune-cell infiltration, and systemic antitumor effects, including enhanced responses to checkpoint inhibitors and abscopal effects in untreated lesions in preclinical models [88].

Emerging preclinical evidence suggests that histotripsy may reduce tumor hypoxia, enhance CD8+ T-cell trafficking, and produce dose-dependent immune effects [89,90,91]. Early human studies have also reported systemic immune activation and possible responses in untreated lesions following liver histotripsy [54,57]. However, these observations do not establish a reproducible clinical abscopal effect. At present, there is no prospective human evidence supporting treatment of only a portion of multifocal hepatic disease in anticipation of immune-mediated control of untreated tumors. The proposed synergy between histotripsy and immunotherapy therefore remains experimental and requires prospective clinical validation. Future trials should evaluate histotripsy in combination with immune checkpoint inhibitors and other systemic therapies while investigating treatment sequencing, immune biomarkers, and whether observed biologic immune responses translate into meaningful clinical outcomes.

#### 5.2.2. Combination with Locoregional Therapies

Histotripsy may also complement established locoregional therapies (LRTs), including transarterial chemoembolization (TACE), transarterial radioembolization (TARE), microwave ablation (MWA), radiofrequency ablation (RFA), cryoablation, and stereotactic body radiation therapy. Although clinical data are limited, combination LRT approaches have improved outcomes in several clinical settings, including intermediate-size hepatocellular carcinoma treated with TACE plus thermal ablation, unresectable liver tumors managed with multimodality locoregional therapy, and oligometastatic disease where local therapies are combined to enhance tumor control, providing a rationale for multimodality strategies involving histotripsy [92,93].

Because histotripsy offers noninvasive focal tumor destruction without heat-sink limitations, it may serve as a consolidative treatment after TACE or TARE, target residual tumor identified on follow-up imaging, or complement thermal ablation in anatomically challenging locations. Future studies should determine optimal sequencing, safety, and efficacy of combined approaches across different treatment intents, including curative, bridging/downstaging, and palliative settings.

### 5.3. Boiling Histotripsy and Technical Advances

Boiling histotripsy is a related focused ultrasound technique that uses shock-wave-induced vapor bubbles to mechanically fractionate tissue [43,94,95]. While current clinical experience is largely based on cavitation cloud histotripsy, boiling histotripsy remains an active area of preclinical development [96]. Animal studies have demonstrated feasibility in liver tissue while highlighting challenges such as respiratory motion, rib obstruction, and acoustic aberration [97,98]. Additional preclinical work suggests potential applications beyond tumor ablation, including treatment of liver fibrosis [96].

Technical advances will be critical for broader adoption of histotripsy. Priorities include improved treatment planning, respiratory motion management, real-time monitoring, automated segmentation, dose optimization, and standardized imaging-response assessment. Strategies such as high-frequency jet ventilation and motion-tracking systems have shown promise for improving targeting accuracy and treatment precision [99,100].

Dose optimization is another important area of investigation. Preclinical studies suggest that treatment dose may influence both local tumor destruction and immune signaling. However, a clinically effective immunomodulatory dosing strategy has not been established in humans, and intentional partial treatment or dose reduction for the purpose of immune priming should not currently be considered an alternative to complete tumor treatment outside a clinical trial. Future prospective studies may determine whether specific treatment parameters produce reproducible immunologic effects and whether these effects have clinical therapeutic relevance.

Overall, future research should focus on prospective clinical validation, integration with multidisciplinary cancer care, and biologically informed combination therapies. Real-world studies such as BOOMBOX, together with investigations of immunotherapy combinations, multimodality LRT approaches, and technical refinements, will help define the long-term role of histotripsy in liver tumor management.

## 6. Conclusions

In conclusion, histotripsy represents an emerging noninvasive and nonthermal liver-directed therapy with high technical success across early prospective and real-world studies. Initial clinical experience in HCC, cholangiocarcinoma, colorectal liver metastases, and other metastatic tumors has demonstrated encouraging short-term imaging and local treatment outcomes, but the evidence base remains early and predominantly noncomparative. Importantly, expanding post-market experience has identified potentially serious complications including portal vein thrombosis, arterial injury with hemorrhage or pseudoaneurysm formation, acute kidney injury, and infection, demonstrating that noninvasive treatment should not be considered risk-free. Prospective studies with standardized imaging assessment, longer oncologic follow-up, systematic complication reporting, and comparative evaluation against established liver-directed therapies are needed to define appropriate patient selection, risk-mitigation strategies, and the long-term role of histotripsy in the multidisciplinary treatment of primary and metastatic liver tumors.

## Data Availability

No new data were created or analyzed in this study.
